# Obituary Craig R. Pringle (1930-2015)

**DOI:** 10.1007/s00705-015-2725-3

**Published:** 2015-12-22

**Authors:** Andrew J. Easton

**Affiliations:** School of Life Sciences, University of Warwick, Gibbet Hill Road, Coventry, CV4 7AL UK

Professor Craig Pringle, Emeritus Professor of Virology at the University of Warwick, died suddenly and unexpectedly at home on Saturday 24 September 2015. Craig was born in Glasgow in 1930, and his early academic achievements led to his parents’ expectations that their only child would follow a career in medicine. However, his interests lay firmly in the more fundamental aspects of science, and he undertook a degree in zoology and genetics at the University of Glasgow, followed by a PhD in microbial genetics at the University of Edinburgh. This early passion for genetics remained with him throughout his scientific career, which focused subsequently on virology. After completing his PhD in 1957, Craig undertook a two-year period of compulsory military service, which he spent at the University of Cambridge using his skill in the Russian language at the Joint Services School for Linguistics. This expertise was also utilised subsequently in undertaking translation of two significant scientific texts and many scientific articles into English. It was typical of Craig’s understated and quiet manner that many who knew him were unaware of his linguistic achievements.

Craig’s first steps in forging an extremely successful career began by picking up his work in genetics at the University of Aberdeen, but the most significant step was his subsequent appointment to the Animal Virus Research Institute at Pirbright (now the Pirbright Institute), where he began to work on the genetics of foot-and-mouth disease virus, ultimately becoming Head of the Department of Virus Genetics at the Institute. His time at Pirbright from 1961 to 1968, interrupted by a very brief return to Aberdeen as a lecturer in bacteriology and particularly by his collaborations with John Subak-Sharpe, set Craig on the path that he followed for the remainder of his career and beyond. In 1968, he moved to the UK Medical Research Council Virology Unit in Glasgow, where John Subak-Sharpe was the newly appointed Director. It was here that Craig undertook most of the work that forged his international reputation for seminal observations on a number of virus systems, particularly vesicular stomatitis virus and respiratory syncytial virus. Craig’s final position was as Professor of Virology in the Department of Biological Sciences at the University of Warwick from 1983 to 1996, where he became an Emeritus Professor upon retirement.

Craig made many pioneering contributions to the field of virology. His careful and meticulous approach in the generation and analysis of virus mutants led to many fundamental observations in the systems he studied. At this time, the use of virus mutants provided the major tool available to virologists for analysis of virus protein functions. Craig utilised his skills and enthusiasm for virology in the exploration of a wide range of different viruses, bringing to each his attentive and critical approach. In particular, his work on vesicular stomatitis virus and other rhabdoviruses laid a foundation for understanding the genetics of these agents. Craig’s work on these and other viruses, including the bunyaviruses, provided unique and invaluable reagents, which he shared generously and without restriction with colleagues across the world. Subsequently, Craig turned his attention to the genetics of pneumoviruses, especially respiratory syncytial virus, and this became the primary focus of his work in the later years of his career. As with all of his areas of interest, Craig developed an international reputation for the quality of this work. Throughout his career, Craig retained a broad interest in virology, and it was a particular pleasure for him to be awarded a Visiting Professorship in the Department of Microbiology at the University of Alabama at Birmingham from 1995 to 1996. During this time, he worked in the laboratory of Gail Wertz, where he returned to his early passion of vesicular stomatitis virus to learn how to manipulate virus genomes using the newly described techniques of reverse genetics. This was a highly successful time, and was a fitting conclusion to a career that had begun by making virus mutants using the traditional approaches of chemical mutagenesis. During his career, Craig was supervisor and mentor to a large number of postgraduate and postdoctoral colleagues. His positive influence is obvious through the successful careers that many of these have had in science since working with him.

Craig’s passion for all things virological continued throughout his retirement from active research, with contributions through work with ProMED (http://www.promedmail.org) and the International Committee on Taxonomy of Viruses (ICTV; http://www.ictvonline.org). He served the ICTV in various capacities, including periods as the *Paramyxoviridae* Study Group chair, a member of the Vertebrate Virus subcommittee, a member of the Executive Committee, and ICTV Secretary – the latter for nine years prior to his retirement. He was also the editor of Virology Division News in Archives of Virology from 1996. He continued to contribute to the work of the ICTV in retirement, with his last published article appearing in 2012.

To all who knew him, Craig was a shy, quiet and thoughtful person, who made light of the many accolades that he received for his work, if he mentioned them at all. He contributed to science at many levels, both directly through his research and also through his contributions to international bodies such as the World Health Organisation. The quality of his contributions to science is clear from his election to life membership of the ICTV in 1999 and to life membership of the UK Society for General Microbiology (now the Microbiology Society) for his work as an editor of the Journal of General Virology from 1982 to 1987.

When away from his research work, Craig’s focus was his family – his wife Judy and three children Kirsty, Iain and Heather. A source of immense pleasure to him was the birth of his first grandchild, Sophia, four months before his untimely death. Outside the laboratory, Craig maintained a keen enjoyment of the remoteness and ruggedness of the Scottish countryside, in particular the Hebridean islands of Colonsay and Jura, where his family spent many holidays. This was a subject that Craig often mentioned, and, after his retirement from Warwick, he returned to Scotland, to the village of Aboyne near Aberdeen. There, he was a keen walker, using his dogs as an excuse to explore the local area while also rekindling an interest in Scottish history and family genealogy. Craig had a rich personal and professional life, with the latter dedicated to the exploration of virology. He will be missed as a good friend, colleague and mentor by all who knew him.

This obituary was previously published at ProMED-mail. Andrew J. Easton, Obituary: Craig Pringle, ProMED Virology Moderator. Archive Number 20151109.3778621, 9 November 2015, http://www.promedmail.org. Accessed 15 December 2015.

